# Supplementary material for: High Pretreatment Serum PD-L1 Levels Are Associated with Muscle Invasion and Shorter Survival in Upper Tract Urothelial Carcinoma
Source: Biomedicines. 2022 Oct 13;10(10):2560. doi: 10.3390/biomedicines10102560 (PMC9599956; doi:10.3390/biomedicines10102560)
Supplement: Supplementary file 1 [file biomedicines-10-02560-s001.zip › biomedicines-1932597-supplementary.pdf]

Supplementary material

High Pretreatment Serum PD-L1 Levels are Associated with Muscle Invasion and Shorter Survival in Upper Tract Urothelial Carcinoma

Ádám Széles [1,2], Petra Terézia Kovács [1], Anita Csizmarik [1], Melinda Váradi [1], Péter Riesz [1], Tamás Fazekas [1,2], Szilárd Váncsa [2,3,4], Péter Hegyi [2,3,4], Csilla Oláh [5], Stephan Tschirdewahn [5], Christopher Darr [5], Ulrich Krafft [5], Viktor Grünwald [5], Boris Hadaschik [5], Orsolya Horváth [6], Péter Nyirády [1] and Tibor Szarvas [1,5,*]

1 Department of Urology, Semmelweis University, Ulloi ut 78/b, 1082 Budapest, Hungary
2 Centre for Translational Medicine, Ulloi ut 26, 1085 Budapest, Hungary
3 Institute for Translational Medicine, Medical School, University of Pécs, Szigeti út 12., 7624, Pécs, Hungary
4 Division of Pancreatic Diseases, Heart and Vascular Center, Semmelweis University, Budapest, Hungary
5 Department of Urology, University of Duisburg-Essen and German Cancer Consortium (DTKK)-University Hospital Essen, Hufelandstraße 55, D-45147 Essen, Germany
6 Department of Genitorurinary Medical Oncology and Clinical Pharmacology, National Institute of Oncology, Ráth György utca 7-9., 1122 Budapest, Hungary
* Correspondence: szarvas.tibor@med.semmelweis-univ.hu

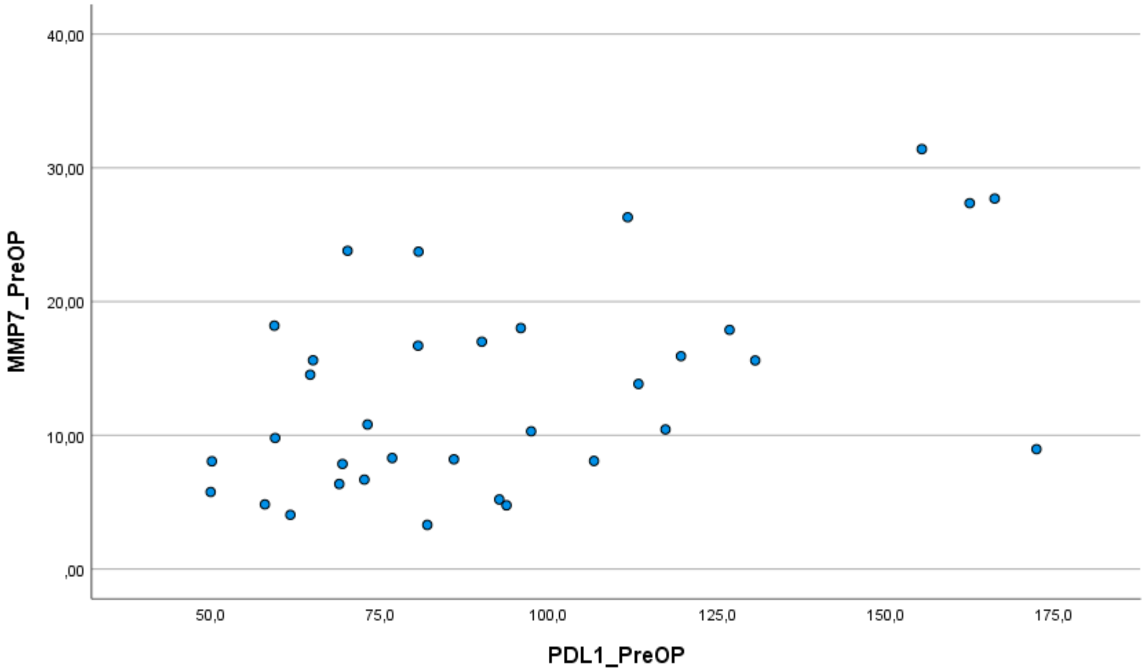

Figure S1. Spearman's rank correlation analysis between preoperative sPD-L1 and MMP-7 levels in the RNU cohort. (rs=0.445, p=0.008)

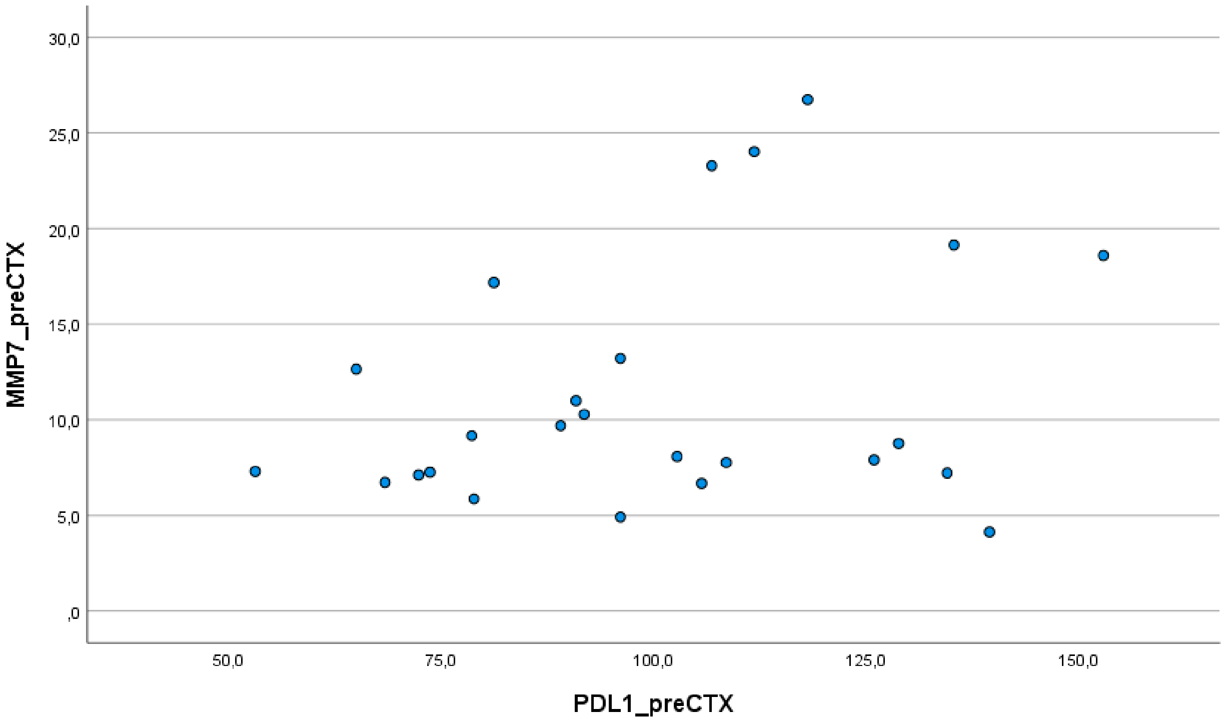

Figure S2. Spearman's rank correlation analysis between pretreatment sPD-L1 and MMP-7 levels in the CTX cohort**.** (rs=0.217, p=0.297)

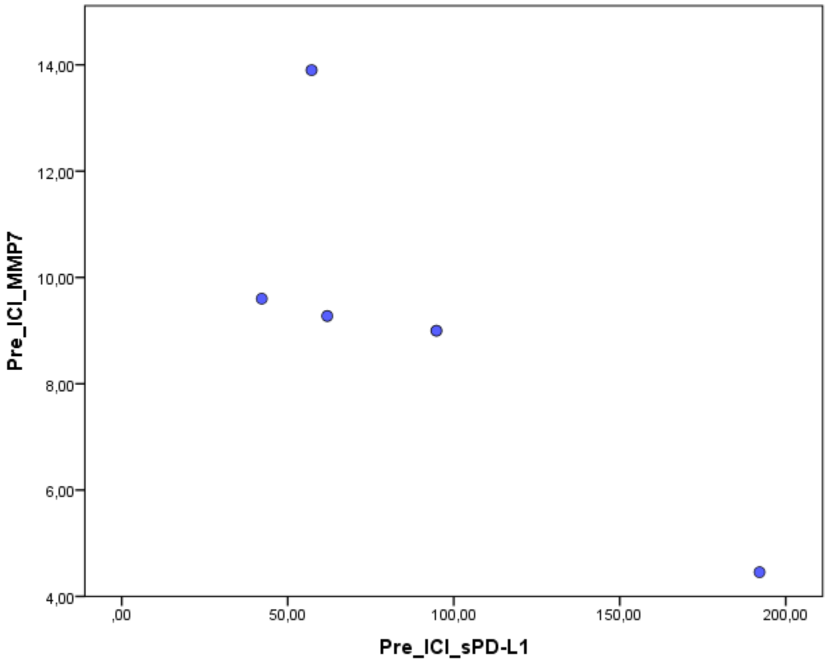

Figure S3. Spearman's rank correlation analysis between pretreatment sPD-L1 and MMP-7 levels in the ICI cohort**.** (rs=0.900, p=0.307)

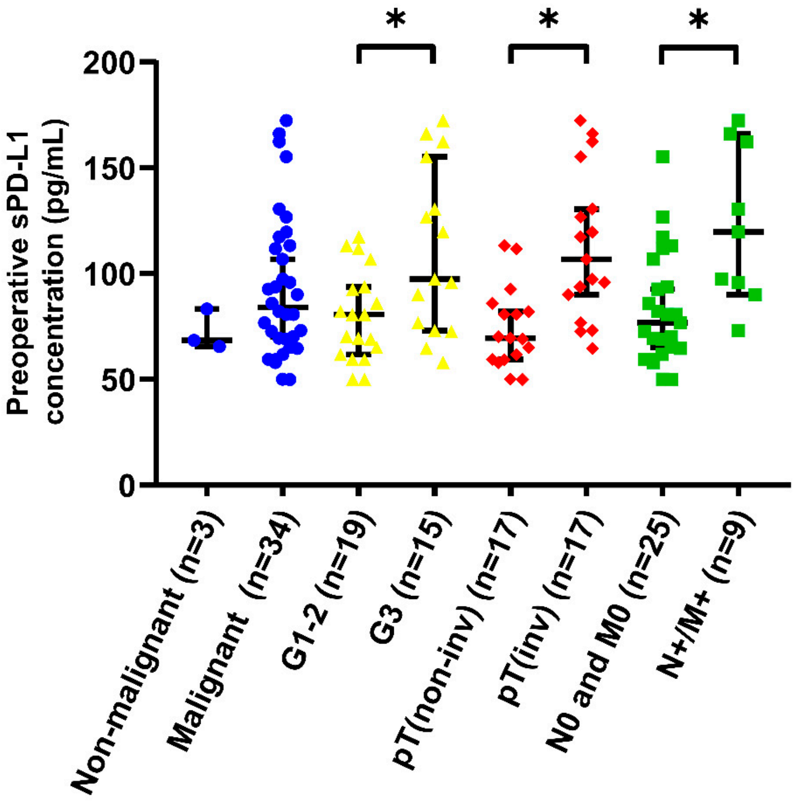

Figure S4. Scatter plot of the association of preoperative sPD-L1 concentration and clinicopathological parameters in the RNU cohort. * significant difference, pT(non-inv): pTa-pT1, pT(inv): pT2-pT4.

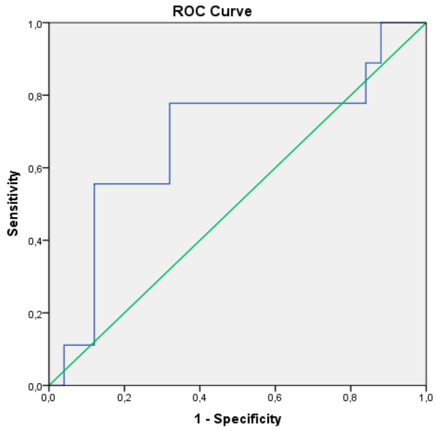

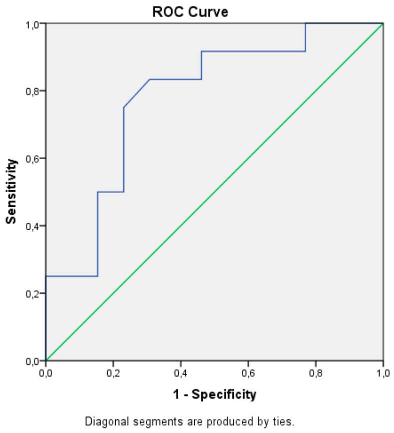

Figure S5. Reciever operating curve (ROC) analysis of the cut-off levels in the RNU and CTX cohorts. RNU cut-off : 118.5 pg/ml (Sensitivity: 0.556, 1-Specificity: 0.120), CTX cut-off: 93.9 pg/ml (Sensitivity: 0.833, 1-Specificity: 0.308),

			RNU							CTX					
			OS			PFS				OS			PFS		
**General data**		**n**	**HR**	**95% CI**	**p**	**HR**	**95% CI**	**p**	**n**	**HR**	**95% CI**	**p**	**HR**	**95% CI**	**p**
Age	≤ 65	10	ref.			ref.			5	ref.			ref.		
	> 65	24	2 .142	0.459 - 9.994	0.332	1.512	0.485 - 4.710	0.476	20	1.548	0.338– 7.094	0.574	0.461	0.157-1.350	0.160
Sex	male	21	ref.			ref.			21	ref.			ref.		
	female	13	0.281	0.060 - 1.301	0.104	0.376	0.120-1.179	0.093	4	0.426	0.055 - 3.310	0.415	0.322	0.042-2.450	0.274
ECOG PS	0-1	29	ref.			ref.			21	ref.			ref.		
	2-3	5	3 .451	0.707 - 16.832	0.126	3.153	0.868 - 11.451	0.081	4	2.490	0.655 – 9.465	0.181	0.931	0.209 - 4.151	0.925
Localization	Ureter	17	ref.			ref.			13	ref.			ref.		
	Pyelon	10	0.927	0.221 – 3.889	0.918	1.713	0.573 – 5.120	0.336	12	2.437	0.730 – 8.143	0.148	0.706	0.248 – 2.010	0.515
RNU data															
Stage	pTa-pT1	17	ref.			ref.				-	-	-	-	-	-
	pT2-pT4	17	7 .115	1.504 - 33.659	**0.013**	10.836	2.865 - 40.978	**<0.001**		-	-	-	-	-	-
Metastases	N0/M0	25	ref.			ref.			14	ref.			ref.		
	N+ or M+	9	4 .891	1.379 - 17.345	**0.014**	6.185	2.199 - 17.397	**0.001**	9	3.948	1.096-14.221	**0.036**	3.470	1.209 - 9.959	**0.021**
CTXbaseline data															
Metastases	N0/M0		-	-	-	-	-	-	10	ref.			ref.		
	N+ or M+		-	-	-	-	-	-	14	14.737	1.810-119.98	**0.012**	7.638	2.218-26.301	**0.001**
CTX regimen	Gem/Cis		-	-	-	-	-	-	14	ref.			ref.		
	Gem/Carb		-	-	-	-	-	-	11	1.077	0.341- 3.401	0.899	1.738	0.623 - 4.846	0.291
Pretreatment sPD-L1															

median cut-off*	17	ref.			ref.			11	ref.			ref.		
median cut-off*	17	4.023	1.060 - 15.269	**0.041**	2.793	1.011 - 7.716	**0.048**	14	6.956	1.461 - 33.110	**0.015**	1.584	0.560 - 4.478	0.386
ROC cut-off**	27	ref.			ref.			11	ref.			ref.		
ROC cut-off**	7	12.114	2.990 - 49.082	**<0.001**	6.667	2.140 - 20.764	**0.001**	14	6.956	1.461 - 33.110	**0.015**	1.584	0.560 - 4.478	0.386

Table S1. Correlation of clinicopathological parameters and pretreatment sPD-L1 concentrations with patients' prognosis * - median cut-off value for RNU is 84.0 pg/ml, median cut-off value for CTX is 96.1 pg/ml; ** ROC cut-off value for RNU is 118.5 pg/ml, ROC cut-off value for CTX is 93.9 pg/ml; RNU – radical nephroureterectomy, CTX – chemotherapy; OS – overall survival; PFS – progression-free survival; ECOG PS – Eastern Cooperative Oncology Group performance status, Gem/Cis – gemcitabine + cisplatin; Gem/Carbo – gemcitabine + carboplatin; bold font represents significant value.
